# Editorial

**Published:** 1873-07

**Authors:** 


### Editorial.

### AMERICAN DENTAL SOCIETY.

The thirteenth annual meeting of the American Dental Association will be held at Put-in Bay—an island in Lake Erie, twenty miles from Sandusky, and forty miles from Toledo—commencing Tuesday, August 5th, at 10 A. M.

ROUTE.

Steamboats leave Detroit for Put-in-Bay at 9 A. M., arriving at 2 P. M. Boats also leave Toledo, at 8 o'clock daily, for Put-in-Bay. Passengers from either the West or East on the Lake shore and Michigan Southern Railroad, or from any of the roads terminating or passing through Sandusky, can take boats for the island, at 9 A. M., and 3, and 7:30 P. M. Passengers on the Baltimore and Ohio Road arrive at Sandusky at 6:30 P. M., in time for the 7:30 boat. M. S. DEAN,

*Secretary.*

We trust that the notice given above by the Secretary of the Association will recive attention by all our readers. And in addition we will say that arrangements have been made for round trip tickets from Cincinnati to Put-in-Bay for $8. All who can come by way of Cincinnati can avail themselves of this rate by applying by letter or otherwise to the Editor of the REGISTER, or to Dr. H. A. Smith, of this city. The route will be by the Hamilton & Dayton R. R., via Toledo ; taking the train at the Hamilton & Dayton Depot at 9 P. M., Monday, Aug. 4th, arrive at Toledo, at 7 o'clock next morning, and take the boat at 8 o'clock A. M., and arrive at Put-in-Bay at 11 o'clock A. M., having a delightful run of forty miles along the most beautiful islands of Lake Erie.

At Sandusky boats connect with trains morning and afternoon for Put-in Bay.

Arrangements have been made by which ample accomodations will be made for all the members of the Dental Association, and their friends who may accompany them. In order however, to make sure of comfortable quarters, every one should notify the proprietors of the Put-in-Bay House, or the proprietor of the Beebe House, of their wishes in this direction, stating the kind of rooms and the length of time they want them, as there will at that time undoubtedly be a very large number of visitors upon the Island.

The fare at the hotels, for all attending the Association, will be $2 per day. We trust that upon this occasion there will be such an outpouring of the dental profession as has not been seen for years ; to this there are many inducements ; the beauties of the place render it one of the most attractive to be found anywhere in the country. The consideration which the proprietors of the hotels have manifested towards the members of our profession not only prospectively, with respect to their meeting, but for several years past to other societies of our profession, is an additional inducement to go now. Let all go who can.

### TRICHLORACETIC ACID.

This acid, which is one of the best substances known for cauterizing warts, corns, etc., may be conveniently prepared by mixing chloral hydrate with its own weight of strong nitric acid, and exposing to sunlight for three or four days The product is distilled, and the portion coming over at about 195° C. is collected apart.

### OHIO DENTAL COLLEGE.

The next regular session of this institution, will begin on Wednesday the 15th of October next.

At which time it is desirable that those who intend to enter the institution, should be present. The chairs are filled by the members of the faculty of last year, except those of Clinical, and Mechanical Dentistry. To the former Dr. J. I. Taylor has been appointed, and to the latter Dr. F. A. Hunter, both of Cincinnati. It is the determination of all interested, to make each succeeding session more thorough and efficient than any preceding, if possible. The state of the profession, and the wants of the public, demand constant progress, and anything that contemplates either a stand still or a retrograde movement is greatly to be regretted. All the ability and facilities of the College, will be devoted to the best interests of those who place themselves in its care.

There is a strong call to all who propose to enter the profession, to make the most thorough preparation, and to accomplish this, the most persevering application and industry is necessary.

### DENTAL MEETINGS.

It was recently our privilege to attend the joint meeting of the Indiana and Kentucky State Dental Societies, held at Louisville, June 4th and 5th. Quite a goodly number of each Society was in attendance, and very interesting sessions were held, nearly all present taking part in the proceedings.

It is a matter of great interest to note the growth and progress of the working societies of our profession, all over the country. The times of prejudice, selfishness, exclusiveness, and self-aggrandizement, are passing rapidly away, and many are pursuing a course of study and investigation in science and art from higher motives. This is true in all legitimate avocations and pursuits; but we see it more clearly in our profession, and nowhere to better advantage than in associations. The desire to learn and know the truth is increasing;—to know it for its own value, and not for some ulterior motive. Union meetings of local societies is an excellent idea,—one that can often be employed to great advantage,—affording opportunities for comparison of ideas, modes of practice, and opinions, that cannot be obtained in small local societies. This suggestion must not be regarded as a discouragement to local societies. These ought everywhere to abound and so cultivated as to yield abundant fruit.

### MICROSCOPIC OBJECTIVES.

Mr. E. Gundlach, the famous maker of microscopic object glasses, formerly of Berlin, Prussia, now of Hackensack, New Jersey, has just completed for us two objectives, a 1-6th and 1-24th immersion. Dr. J. J. Woodward, the distinguished microscopist of the Army Medical Museum at Washington, to whom we submitted them for examination, in a letter to us speaks of their performance in the most complimentary manner, saying: "I am sorry you were not present at my trial of the objectives, for I think you would have been pleased with their performance. The 1-6th did admirably on amphipleura pellucida (by sunlight), and is, I think, correctly named. At 50 inches from micrometer to screen it magnified 300 diameters, when corrected for uncovered objects; 340 when the collar was fully closed. The 1-24th showed the nineteenth band of Nobert, and resolved amphipleura superbly. It is not correctly named according to the English nomenclature; at 50 inches from micrometer to screen it measures 850 diameters at the uncovered point; 1150 when fully closed. It is therefore properly about a 1-17th. I suppose Gundlach named it by its performance when closed, which is equivalent to that of a single lens, of 1-23d focal length; but that is not the way we do in this country and England.

"When the price is taken into consideration, both of these objectives are wonderfully good."

Wales' 1-15th, as tested by Dr. Woodward, and reported by Dr. Richardson, of Philadelphia, in his work on Medical Microscopy, resolves only the seventh band of Nobert.

The English, as is well known, are in the habit of underrating the magnifying power of their objectives ; Powell & Lealand's 1-16th, for example, is more properly a 1-20th. Mr. Carpenter in his work on microscopy speaks of this disposition on the part of English makers to rate their objectives at less than they should be, as, for instance, he states, a power that is usually a fifth is named a quarter, etc. ; for it is to a manufacturer's credit for a quarter to do the work of a fifth, or for any lower power to do that which ordinarily could only be expected by a higher power. Mr. Gundlach, however, in his mode of measurement in rating the power does not do his lenses justice when compared with those of American and English manfacture.

In our own hands the performances of the objectives made for us by Mr. Gundlach are fully equal to those of the most celebrated makers of the world. In the few days we have been in possession of them, we have been using them in resolving diatoms, of which we have a very large number by different mounters ; and we have had no difficulty in the resolution of the most difficult tests with either the 1 24th or 1-6th. We have a Powell & Lelands 1-16th immersion, of which Mr. Powell said that a better one had never left their manufactory, and up to the present time we have been able to resolve, with either one of the lenses which Gundlach has made for us, all that we have been able to do with it. A 1-6th that resolves "admirably" the markings of amphipleura pellucida, a test that is considered by some, if any thing, more difficult than the nineteeth band of Nobert, must hold the highest place among objectives of the finest definition.

A matter, too, of consideration with the majority of persons in the purchase of Gundlach's objectives is their great cheapness. The price of his 1-6th is only $26 (gold). The price of Tolles' 1-6th, of a like angle of aperture, is $75 (currency). Gundlach's 1-24th is quoted at $55 (gold) ; Tolles' 1-20th at $190 (currency).

We regard an objective that is able to resolve in a most satisfactory manner all the most difficult tests found among the diatoms, which Gundlach's can do, the price of which is only about $30 currency, as one of the remarkable things.—*Cin. Med. News.*

### THIRD DENTITION.

Lee R. McFarland, of Bedford, Mich., has met with a case of third dentition—" a second molar on each side, one bicuspid and a canine."

These unbidden guests, it appears, made their advent under a well fitting artificial denture, and not only upset the denture and completely overturned the expectations of the patient, but put to shame the representations of the practitioner whose "plate wouldn't fit." He asks: "Will the patient have any more? Would it be good practice to extract those he has al ready?"

### DIAGNOSIS.

The difficulty of detecting the beginning of decay on proximal surfaces of teeth that are firmly crowded together is often very great. This is sometimes effected by wedging apart, and occasionally the color will prove a sufficient indication. If there be not time for the former and the latter fail, another method may be employed, which I believe was first proposed by Henry S. Chase. Force linen thread between the teeth. If it is cut or very much roughened while passing between the bodies of the teeth and below the grinding surfaces, there is a roughened and abraded enamel surface.

### RECOVERING SILVER FROM RESIDUES.

J. Kruger recommends to shake up the argentiferous liquors with phosphoric ether (phosphorus dissolved in ether) until the precipitate produced is of a uniform black color. The precipitate is then separated from the mother liquor, washed, and boiled with caustic potash solution. Pure metallic silver is the result.

### A NEW THING.

Dr. J. W. Baxter, of Vevay, Ind., has invented and constructed a machine for cutting Arkansas stone into small wheels, cylinders, and cones—indeed, into any desirable form for dental purposes: and such is its perfection, that the work is done rapidly and very accurately.

They are mounted upon mandrels, and are used in the Morrison Lathe, and may be mounted for any other lathe. They are very valuable indeed, and for a variety of purposes, which will readily be suggested to the mind of the skillful operator. Of the uses to which they are so well adapted, we find none more effective than that of cleaning the teeth—removing the deposit which so greatly discolors them, adheres firmly, and is so difficult to remove by the ordinary means. These little Arkansas stone cones are, for the purpose, more efficient than anything we have ever used. They should be in the hands of every dentist. Dr. Baxter will doubtless have them in the market soon.

He also makes a diamond point burnisher, from what is known as the Alaska diamond, that is far superior to any steel brusher. No one after having become familiar with the use of these things, would be content to be without them.

### NICKEL.

We see by a paragraph in a foreign scientific journal that the manufactures of German silver have presented a petition to the Reichstag against the introduction of a nickel coinage in the German Empire. They urge that the price of nickel has recently risen from 1½ to 4 thalers (the thaler is worth about 70 cents, gold), and that German silver has in a single month advanced 8 thalers per hundredweight. In England, as we learn from other sources, it has gone up, within a year and a half, from 4 shillings to 16 shillings a pound.

It is only within a few years that this metal has become of much importance in the useful arts, and it appears that already the demand for it exceeds the supply. The total annual production amounts to only about 600 tons, and full one-half of this is required for manufacturing purposes in England alone. It is now employed for coinage in Belgium, Switzerland, and the United States, and if it should be adopted in the German Empire, the amount absorbed in this way would soon interfere seriously with its application to industrial uses. If it were abundant there is no doubt that it would be far more extensively employed in the arts. Its tensile strength is almost one third greater than that of iron, to which metal it bears a marked analogy in most of its properties, and it is also less liable to rust in air or water. German silver is simply a brass to which from one-sixth to one-third of nickel has been added, giving it a silvery white color, and at the same time greatly increasing its power of resisting chemical agents. As a foundation for electrotyping with silver, it has the advantage over copper of not showing a red color upon worn edges. It is also beginning to be used itself for electroplating other metals, and this is likely to become an important branch of manufacture.

The increasing demand for nickel will doubtless stimulate the search for new sources of supply; but until these are discovered it is evidently desirable that the use of the metal for coinage should not be extended. How small the present supply *is* will perhaps be clearer to our readers if we compare it with that of one or two other metals. Of copper, for instance, the annual production is about 65,000 tons, or more than a hundredfold greater; while of iron more than 10,000,000 tons are yearly added to the immense amount absorbed in the industries of the world. On the other hand the annual production of platinum is only from 35 to 50 hundredweight, or an average of about *two* tons.

